# Cancer detection and biopsy classification using concurrent histopathological and metabolomic analysis of core biopsies

**DOI:** 10.1186/gm332

**Published:** 2012-04-30

**Authors:** Meredith V Brown, Jonathan E McDunn, Philip R Gunst, Elizabeth M Smith, Michael V Milburn, Dean A Troyer, Kay A Lawton

**Affiliations:** 1 Metabolon, Inc., 617 Davis Drive, Suite 400, Durham, NC 27713, USA; 2 Eastern Virginia Medical School, 700 W. Olney Rd, Norfolk, VA 23501, USA

## Abstract

**Background:**

Metabolomics, the non-targeted interrogation of small molecules in a biological sample, is an ideal technology for identifying diagnostic biomarkers. Current tissue extraction protocols involve sample destruction, precluding additional uses of the tissue. This is particularly problematic for high value samples with limited availability, such as clinical tumor biopsies that require structural preservation to histologically diagnose and gauge cancer aggressiveness. To overcome this limitation and increase the amount of information obtained from patient biopsies, we developed and characterized a workflow to perform metabolomic analysis and histological evaluation on the same biopsy sample.

**Methods:**

Biopsies of ten human tissues (muscle, adrenal gland, colon, lung, pancreas, small intestine, spleen, stomach, prostate, kidney) were placed directly in a methanol solution to recover metabolites, precipitate proteins, and fix tissue. Following incubation, biopsies were removed from the solution and processed for histology. Kidney and prostate cancer tumor and benign biopsies were stained with hemotoxylin and eosin and prostate biopsies were subjected to PIN-4 immunohistochemistry. The methanolic extracts were analyzed for metabolites on GC/MS and LC/MS platforms. Raw mass spectrometry data files were automatically extracted using an informatics system that includes peak identification and metabolite identification software.

**Results:**

Metabolites across all major biochemical classes (amino acids, peptides, carbohydrates, lipids, nucleotides, cofactors, xenobiotics) were measured. The number (ranging from 260 in prostate to 340 in colon) and identity of metabolites were comparable to results obtained with the current method requiring 30 mg ground tissue. Comparing relative levels of metabolites, cancer tumor from benign kidney and prostate biopsies could be distinguished. Successful histopathological analysis of biopsies by chemical staining (hematoxylin, eosin) and antibody binding (PIN-4, in prostate) showed cellular architecture and immunoreactivity were retained.

**Conclusions:**

Concurrent metabolite extraction and histological analysis of intact biopsies is amenable to the clinical workflow. Methanol fixation effectively preserves a wide range of tissues and is compatible with chemical staining and immunohistochemistry. The method offers an opportunity to augment histopathological diagnosis and tumor classification with quantitative measures of biochemicals in the same tissue sample. Since certain biochemicals have been shown to correlate with disease aggressiveness, this method should prove valuable as an adjunct to differentiate cancer aggressiveness.

## Background

The gold standard for diagnosis and staging of many diseases is histopathology. Grading systems have been developed to predict tumor aggressiveness, and the pathologist's report often guides clinical treatment decisions. However, all grading systems are subjective. Intra- and inter-observer variability occur frequently as exemplified in renal cell carcinoma, prostate cancer, and bladder cancer [1-6]. Discordance between biopsies and resected specimens also occurs [5,7]. The development of molecular analytic techniques such as immunohistochemistry and fluorescence *in situ *hybridization (FISH) has enhanced microscopic examination and has allowed for biomarker discovery. Both techniques have been widely adopted into the practice of pathology [8,9]. More recently, high-throughput molecular analytic techniques for non-targeted RNA, DNA and protein determinations have been introduced. Complementary to those approaches is metabolomics, the process of cataloging and quantifying the low molecular weight (<1,500 Da) components of biologic material. Recent reports have demonstrated that metabolomics can also reveal disease-specific signatures that have the potential to aid in disease diagnosis and management [10-14]. While there are many efforts underway to discover and implement metabolomic biomarkers in blood and urine, tissue remains a major focus for biomarker discovery and implementation.

Historically, tissue metabolomics has been performed using large pieces of tissue (>30 mg). In order to achieve rapid, complete metabolite extraction, the tissue was ground, destroying the cellular and tissue architecture that are critical for pathological assessment, including immunohistochemistry and FISH. These limitations largely prevented the use of metabolomics for evaluating clinical biopsies. Using a method of biopsy incubation in aqueous alcohol [15], we describe and characterize a novel workflow that overcomes these limitations and can be implemented in a standard clinical pathology practice. Alcohol has, for many years, been the standard fixative for use in cytology. As a less toxic alternative to formaldehyde, alcohol-based fixation is being increasingly used for routine pathology. Analyzing the same biopsy using both histopathology and metabolic profiles/biochemical biomarkers could increase the accuracy of pathology-based diagnoses. Our results demonstrate that metabolic profiles could augment pathology reports by adding a quantitative biochemical-based metric to the information obtained from patient biopsies.

## Methods

### Sample collection

Human kidney tissue used for method optimization experiments was obtained from nephrectomy patients according to the Eastern Virginia Medical School Institutional Review Board (08-11-WC-0213). Post-nephrectomy, kidney specimens were transported immediately to the pathology suite and sampled. Renal tumor tissue was readily identified and could be separately sampled from the unaffected non-tumor-containing kidney tissue. Normal tissue was flash frozen, stored at -80°C, and processed as indicated below.

Adrenal gland, colon, lung, muscle, pancreas, small intestine, spleen, and stomach tissues were obtained from beating heart donors by LifeNet Health, Transplant Services Division (1864, Concert Drive, Virginia Beach, VA, USA). LifeNet is a federally designated Organ Procurement Organization that coordinates the recovery and transplantation of organs across Virginia, including the city of Norfolk. The consent process for organ donation includes the opportunity for next of kin to separately consent to procurement and use of non-transplantable organs and tissues for research. When such research consent existed, 0.5 to 1 cm^3 ^portions of tissue were obtained following established protocols of the procurement team. Tissue was collected just prior to the withdrawal of support and placed immediately in 80% methanol to fix the tissue for histological analysis and to extract metabolites.

For case/control samples, core biopsies were obtained post-operatively from six renal cancer patients and eight prostate cancer patients. Kidney needle biopsies were obtained from renal tumor tissue and benign kidney tissue post-nephrectomy using an 18 gauge needle and placed directly into methanol. Similarly, needle biopsies of prostate tissue were obtained post-prostatectomy. After weighing and measuring the prostate, prior to inking, the prostate was oriented posterior surface upwards with the apex toward the operator. An 18 gauge biopsy gun was used to acquire 12 cores distributed in a fashion that mimics that utilized for *in vivo *ultrasound-directed core biopsies (one each as left apex lateral, left apex transition, left mid lateral, left mid transition, left base lateral, and left base transition; the process was repeated for the right prostate). The cores were then placed directly into methanol. All samples were collected with informed consent by approval of the Eastern Virginia Medical School Institutional Review Board.

For the incubation time course, fresh frozen normal human kidney tissue was purchased from Asterand (Asterand, Inc., Detroit, MI, USA).

### Sample preparation and metabolite extraction

Following sample collection, a single biopsy was placed directly in a Nalgene cryovial containing 2 ml of solvent (80% methanol, 20% ultra-pure water unless otherwise noted). Samples were incubated for 24 h (unless otherwise noted) at room temperature (22 to 24°C). After a 5 minute spin at 2,000 rpm, the solvent extract was transferred to a clean vial and evaporated to dryness under a stream of nitrogen gas at 40°C in a Turbovap LV evaporator (Zymark, Hopkinton, MA, USA). The dried extracts were reconstituted in 550 μl methanol:water (80:20) containing recovery standards (D,L-2-fluorophenylglycine, D,L-4-chlorophenylalanine, tridecanoic acid, D6 cholesterol). For experiments where histology was performed on the biopsy, the biopsy was removed from the solvent and processed for histology as indicated below.

For optimization experiments using 30 mg tissue pieces, tissue was cut, weighed directly in a vial, and the weight was recorded. To each vial 600 μl of 80% methanol (unless otherwise noted) containing recovery standards were added. The tissues were homogenized in a Geno-grinder 2000 (SPEX, Metuchen, NJ, USA), and the samples were spun for 1 minute at 2,000 rpm. The concentration of the ground extract was adjusted by adding 80% methanol to be equivalent to 32 mg of initial wet weight per milliliter of methanol extract. Samples were mixed, then spun for 5 minutes at 2,000 rpm to pellet any particulate. Volumes of 550 μl (17.6 mg tissue equivalent) of the reconstituted solution were analyzed by metabolomics as described below. For experiments analyzing ground biopsies and post-extraction ground biopsies, 600 μl of indicated solvent containing recovery standards were added to each biopsy. The tissue was homogenized in a Geno-grinder 2000 (SPEX) and spun down for 1 minute at 2,000 rpm. An additional 50 μl of methanol containing recovery standards were added to the samples. They were mixed and spun down for 5 minutes at 2,000 rpm. A 550 μl aliquot of the solution was analyzed by metabolomics.

### Histology

After methanol incubation, the needle biopsies were placed in biopsy bags and cassettes, which were then transferred to Molecular Fixative (UMFix, Sakura, Torrance, CA, USA) until processed for histology. Biopsies were processed on a Tissue-Tek Xpress x50 (Sakura) following the manufacturer's instructions with an approximate run time of 1.5 h. All processing reagents were purchased from Sakura (Sakura-Finetek, Torrance, CA, USA). Briefly, two 15 minute incubations at 40 to 44°C in an acetone/alcohol solution with agitation were followed by a 15 minute incubation at 64 to 66°C in a vacuum in a mineral oil paraffin reagent and finally another 15 minute incubation at 64 to 66°C in a vacuum in a paraffin reagent. Tissues were embedded immediately following processing and were sectioned and placed on slides.

Sections were de-paraffinized and rehydrated by 3 × 3 minute incubations in xylene, 3 × 3 minute incubations in 100% ethanol, 1 × 3 minute incubation in 95% ethanol, 1 × 3 minute incubation in 80% ethanol and 1 × 5 minute incubation in deionized water. Hematoxylin stain was performed by a 3 minute incubation in hematoxylin, a rinse in deionized water, 5 minute incubation in water, and 8 to 12 rapid dips in 0.3% acidified ethanol (2,800 ml ethanol:1,200ml water:12 ml concentrated hydrochloric acid) to de-stain followed by 2 × 1 minute incubations in tap water and a 2-minute rinse in deionized water. Slides were then placed in eosin for 30 s followed by 3 × 5 minute incubations in 95% ethanol, 3 × 5 minute incubations in 100% ethanol, and 3 × 15 minute incubations in xylene. Coverslips were then mounted onto slides using Permount (Fisher Scientific, Waltham, MA, USA). All sections were examined and analyzed by a board-certified pathologist (DAT).

For immunohistochemistry, PIN-4 pre-diluted cocktail (P504S, HMW Cytokeratins, and p63; Cat # PPM 225DS) was purchased from Biocare Medical (Concord, CA, USA). The Ventana BenchMark XT Automated Slide Preparation System (Ventana Medical Systems, Inc., Tuscon, AZ, USA) was used to process the samples.

### Metabolomic profiling

Global metabolomic profiling was carried out on three independent instrument platforms, one gas chromatography/mass spectrometry (GC/MS) and two ultrahigh performance liquid chromatography/tandem mass spectrometry (UHLC/MS/MS^2^) platforms optimized for either basic species or acidic species. Detailed descriptions of these platforms, including instrumentation configurations and conditions, data acquisition, and software approaches for data handling, were previously described in detail [16,17]. The major components of the process are summarized below.

Following metabolite extraction, samples were separated into three equal aliquots, using an automated MicroLab STAR® system (Hamilton Company, Salt Lake City, UT, USA), for analysis on three independent platforms as described below. The samples destined for GC/MS analysis were dried under vacuum desiccation for a minimum of 24 h and then derivatized under dried nitrogen using bistrimethyl-silyl-triflouroacetamide (BSTFA). Samples were analyzed on a Thermo-Finnigan Trace DSQ fast-scanning single-quadrupole mass spectrometer using electron impact ionization. UHPLC/MS/MS^2 ^was carried out using a Waters Acquity UHPLC (Waters Corporation, Milford, MA, USA) coupled to an LTQ mass spectrometer (Thermo Fisher Scientific Inc., Waltham, MA, USA) equipped with an electrospray ionization source. Two separate UHPLC/MS/MS^2 ^injections were performed on each sample: one optimized for positive ions and one for negative ions. Chromatographic separation followed by full scan mass spectra was carried out to record retention time, molecular weight (m/z) and MS/MS^2 ^of all detectable ions presented in the samples.

Metabolites were identified by automated comparison of the ion features in the experimental samples to an in-house reference library composed of more than 3,000 authentic chemical standard entries that included retention time, molecular weight (m/z), preferred adducts, and in-source fragments as well as their associated MS/MS^2 ^spectra. This library allowed the rapid identification of metabolites in the experiment with high confidence.

### Statistical analysis

Missing values for a given metabolite were imputed with the observed minimum detection value based on the assumption that they were below the limits of instrument detection sensitivity. All comparisons were performed using log-transformed data. Welch's two sample *t*-tests were used for all comparisons unless otherwise noted. Multiple comparisons were accounted for with the false discovery rate (FDR) method, and each FDR was estimated using q-values [18]. For convenience of data visualization, raw area counts for each biochemical were re-scaled by dividing the value for a specific biochemical in each sample by the median value for that specific biochemical.

Hierarchical clustering based on Euclidean distances was performed with all metabolites determined to be statistically significant (*P *≤ 0.05) when comparing cancer tumor to benign. Principal components analysis was performed using the correlation matrix from significant metabolites in order to graphically illustrate structure in the metabolomic data. Due to the potential for false positives in this subset of significant metabolites, this approach results in a slight over-fitting of the data. Random forest analysis [19] was used for classification of samples into groups (for example, cancer tumor or benign). Random forests give an estimate of how well individuals in a new data set can be classified into each group, in contrast to a *t*-test, which tests whether the unknown means for two populations are different or not. Random forest analyses were performed to classify cancer tumor and benign samples (prostate and kidney). All statistical analyses were generated using Array Studio software. Array Studio, Array Viewer and Array Server and all other Omicsoft products or service names are registered trademarks or trademarks of Omicsoft Corporation, Research Triangle Park, NC, USA.

## Results and discussion

### Method validation

**Figure 1:**
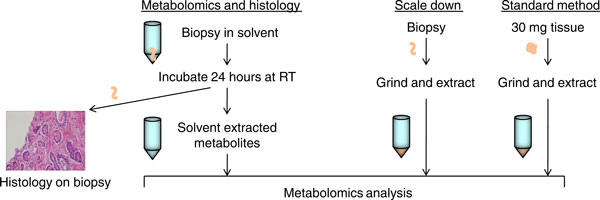
**Schematic outline of method workflow**. Flow diagram of the intact biopsy extraction protocol and tissue grinding protocol for a tissue biopsy and a 30 mg tissue piece. RT, room temperature.

**Figure 2:**
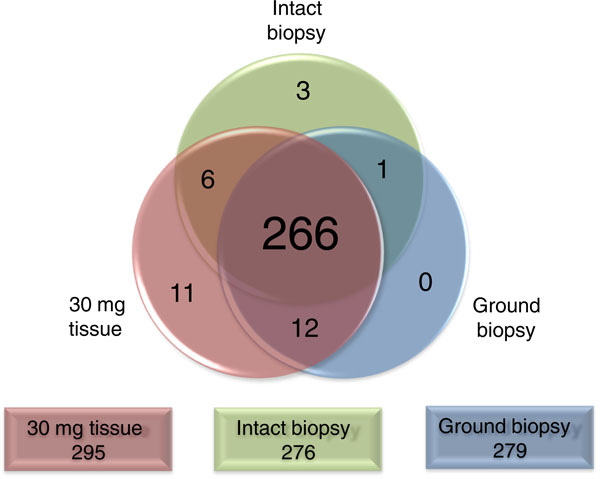
**Number and identity of metabolites obtained with intact biopsy method and the standard ground tissue extraction method**. The total number of metabolites detected using each sampling and extraction protocol (30 mg tissue, intact biopsy, ground biopsy) is shown in the rectangles at the bottom of the figure. The Venn diagram represents the overlap in the identity of metabolites detected using each method. The vast majority (266) of metabolites are detected using all three methods. Metabolites were extracted from intact biopsies with 80% methanol.

To compare this method with current metabolomic extraction techniques, we prepared *ex vivo *biopsy samples (3 to 5 mg of tissue) from freshly collected human kidney. In order to assess the extraction efficiency of the biopsy method, three sampling strategies were compared: intact extracted biopsies, ground biopsies, and 30 mg pieces (Figure 1). We identified 299 metabolites in ground human kidney samples, and >92% of these compounds were also identified in the intact biopsy samples (Figure 2). Thus, despite the ten-fold less tissue and the absence of tissue disruption, the intact biopsy extraction method is comparable to standard metabolomic extraction techniques. We obtained similar results when using 70% methanol (Additional file 1).

To determine the efficiency of intact biopsy extraction, we first extracted metabolites from intact biopsy samples and then ground the tissue and performed a second extraction. Only 143 metabolites were detected following this secondary extraction, and the levels were reduced by an average of 81% (median reduction of 93%) compared to the level in the initial extract (Additional file 2), indicating near complete extraction using this method.

**Figure 3:**
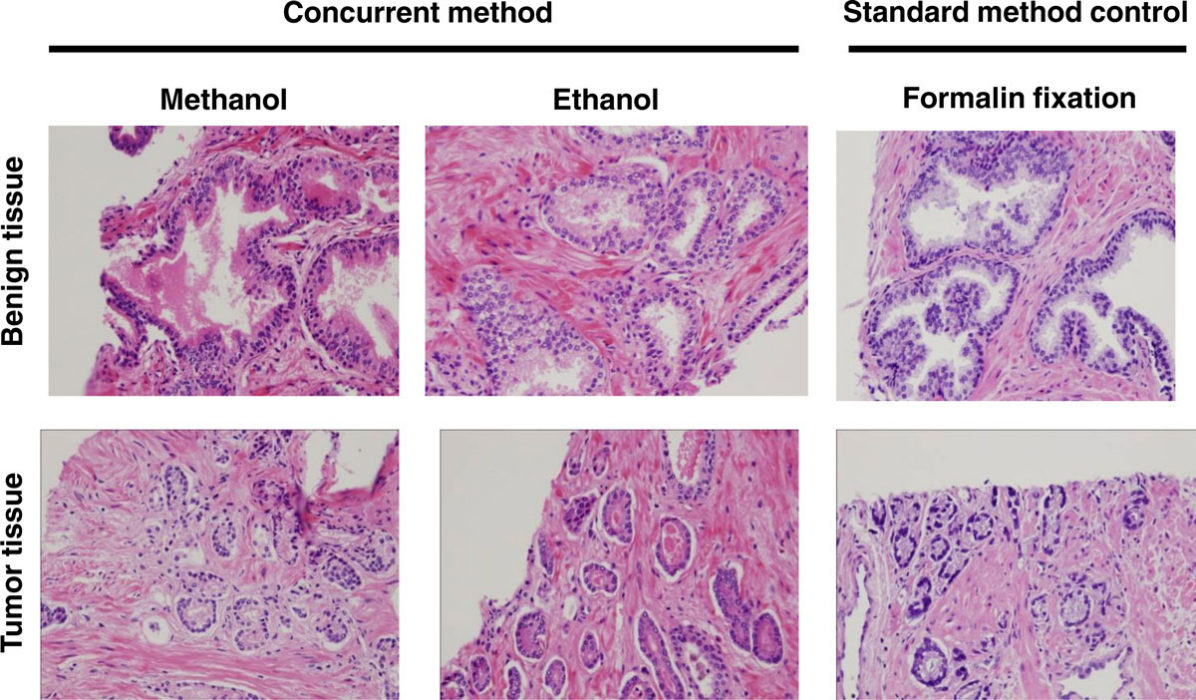
**Histochemical staining of biopsy samples treated with methanol or ethanol as the extraction solvent**. Human prostate biopsies from benign or cancer tumor tissue were processed using the intact biopsy method in either 80% methanol or 70% ethanol or fixed in formalin followed by paraffin embedding and sectioning. The resulting sections were then stained with hematoxylin and eosin.

**Figure 4:**
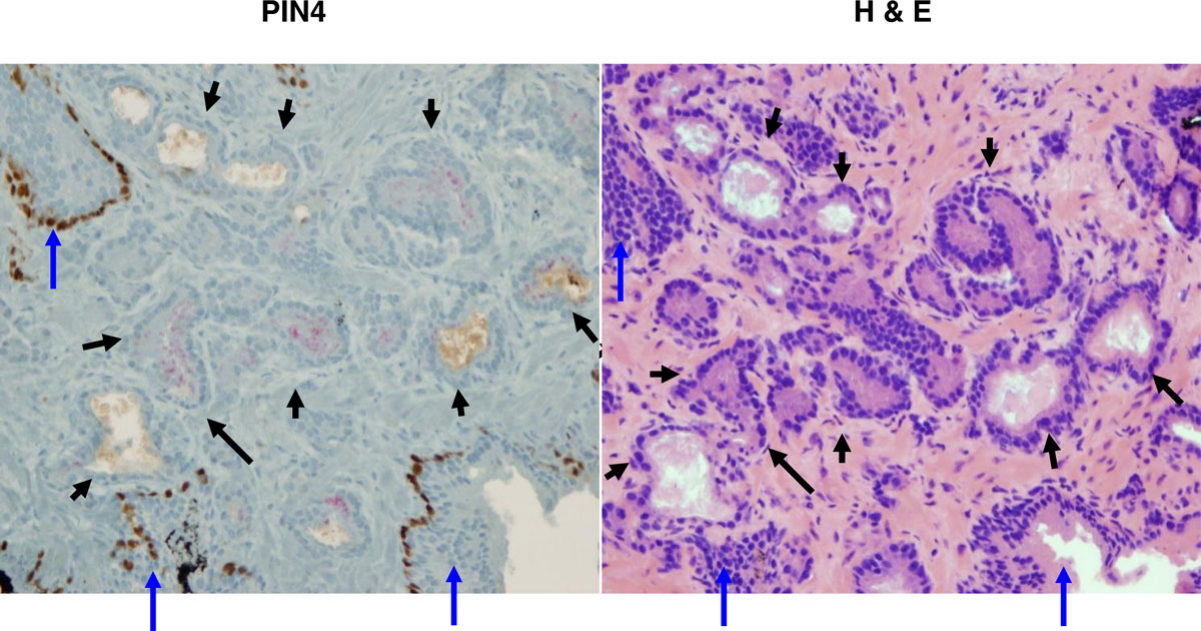
**Histology of prostate biopsy samples**. Human prostate biopsy samples were processed using the intact biopsy method in 80% methanol followed by paraffin embedding and sectioning. **(a) **Prostate section processed for immunohistochemistry using PIN4 stain where red indicates racemase and brown indicates p63 and basal keratin. **(b) **An immediately adjacent section stained with hematoxylin and eosin (H & E). Black arrows indicate prostatic adenocarcinoma and blue arrows indicate benign glands.

To assess the histopathology of post-extraction biopsy tissue, we transferred prostate biopsy tissue from the extraction solvent (methanol or ethanol) directly into Molecular Fixative and followed a formalin fixation workflow. We compared these results with biopsy tissue that was fixed directly in formalin. Hematoxylin and eosin staining revealed minor differences between alcohol and formalin fixed tissues, but the tissue architecture was equally well-preserved in all cases (Figure 3). We also performed immunohistochemical analysis of prostate tissue prepared with this workflow using the PIN4 antibody cocktail. Appropriate staining of prostatic adenocarcinoma and benign glands can be seen (Figure 4), validating a previous observation [15]. This result suggests that antigen retrieval is not substantively altered by the workflow and demonstrates the potential utility of this workflow in standard histology practice.

While we have shown that this method is compatible with one antibody cocktail, we acknowledge that fixation in alcohol is not the standard method and that additional validation may be needed for additional antibodies. We have shown that metabolites can be readily extracted from tissue biopsies by soaking in aqueous alcohol. Formalin fixation protocols include aqueous alcohol incubation steps, so one approach could be to perform metabolomics analysis on the extract from this first alcohol incubation. This modification would allow for tissues to be processed using formalin fixation, minimizing the deviation from standard pathology practice. Studies are ongoing to assess the feasibility of this approach.

### Optimization and implementation of workflow

With the validated method, we sought to test parameters of the workflow. As formalin fixation time can vary by investigator protocol, we performed a time course (ranging from 0.5 to 48 h) to determine the optimal methanol incubation time. Metabolites were extracted within 0.5 h of methanol incubation, and the number (Table 1) and identity (Additional file 3) of the metabolites detected at each time point remained consistent. To assess changes in the levels of metabolites throughout the timecourse, relative metabolite levels at each timepoint were compared to the 24 h timepoint, which was the incubation time used in the method validation studies. The number of significantly altered metabolites for each comparison is presented in Table 2. Differences were seen between the metabolite levels at the shorter (0.5 to 4 h) incubation times compared to the 24 h time point, but these differences were not observed following 8 h incubation (Table 2). Based on these results for kidney tissue, there is no clear optimal incubation time. These data suggest that as long as the incubation time is consistent, this method is amenable to the investigator's protocol.

**Table 1:** Number of metabolites detected following various incubation times

Incubation time (hours)	Metabolites detected
0.5	292
1	293
4	293
8	294
16	293
24	292
48	292

Biopsy samples from normal human kidney tissue were incubated in 80% methanol for 0.5 h, 1 h, 4 h, 8 h, 16 h, 24 h, and 48 h. The extracts were processed for metabolomics analysis. The number of metabolites detected following each incubation time is shown.

**Table 2:** Statistical summary for methanol incubation time course

Summary of altered biochemicals (Welch's two-sample *t*-test)	0.5H/24H	1H/24H	4H/24H	8H/24H	16H/24H	48H/24H
Total number of biochemicals with *P *≤ 0.05	28	50	30	0	0	0
Biochemicals (increased|decreased)	11|17	25|25	18|12	0|0	0|0	0|0
q-value	0.09	0.09	0.10	N/A	N/A	N/A

Biopsy samples from normal human kidney tissue were incubated in 80% methanol for 0.5 h, 1 h, 4 h, 8 h, 16 h, 24 h, and 48 h. The solvent extracts were processed for metabolomics analysis. The summary of the number of biochemicals significantly (*P *≤ 0.05, q ≤ 0.1) altered for the indicated comparison, as determined by Welch's two-sample *t*-test, is shown.

To assess this workflow in the clinical setting, we analyzed a panel of eight tissues obtained from consented donors prior to withdrawal of support (beating heart donors). For all cases, fresh tissue was placed directly in 80% methanol to extract metabolites, then the biopsy was removed for histological processing and analysis. Between 260 and 340 metabolites across all major biochemical classes were measured in the tissues profiled (Table 3; Additional file 4). Hematoxylin and eosin staining shows the tissue architecture was retained (Additional file 5). These results demonstrate the utility of this workflow in an array of tissue types within a clinical setting. Moreover, these metabolomic inventories of histologically normal human tissues serve as a baseline for future studies of both normal human variation and disease-induced alterations in tissue metabolism. In addition, this is the first reported metabolomic catalog of small intestine, adrenal gland and spleen from humans.

**Table 3:** Number of metabolites in the major biochemical classes detected in various human tissues

	Tissue type									
	
Biochemical super pathway	Adrenal gland (1)	Colon (4)	Lung (1)	Muscle (4)	Pancreas (3)	Small intestine (4)	Spleen (1)	Stomach (4)	Prostate (8)	Kidney (3)
Amino acid	69	80	71	77	71	74	62	80	70	81
Peptide	35	40	36	37	42	43	38	42	6	24
Carbohydrate	25	26	27	28	26	25	23	27	21	19
Energy	8	8	8	8	8	8	8	8	10	7
Lipid	102	129	112	122	117	124	90	125	101	107
Nucleotide	23	28	24	25	26	26	24	27	20	22
Cofactors and vitamins	8	12	9	10	8	9	7	10	13	13
Xenobiotics	13	17	12	15	13	15	10	15	17	20
Total	**283**	**340**	**299**	**322**	**311**	**324**	**262**	**334**	**260**	**293**

Adrenal gland, colon, lung, muscle, pancreas, small intestine, spleen, and stomach samples were collected from beating heart donors just prior to the withdrawal of support, placed immediately in 80% methanol and incubated for 24 to 72 h at room temperature. Prostate refers to benign human tissue biopsy samples collected post-prostatectomy from regions of the prostate that did not contain cancer and processed in 80% methanol as described. Kidney refers to the fresh frozen normal human kidney tissue analyzed in the incubation time course experiment reported in Table 1. The number of metabolites detected in each biochemical class is indicated. The number of samples analyzed for each tissue type is indicated in parentheses.

### Clinical application of workflow in disease state

**Figure 5:**
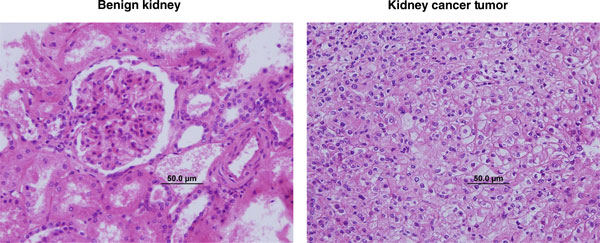
**Representative histology images from kidney biopsies show tissue structure is retained**. Patient-matched **(a) **benign and **(b) **cancer tumor kidney biopsies were processed using the intact biopsy workflow and stained with hematoxylin and eosin. Scale bars, 50 μm.

To demonstrate the utility of the workflow in a clinical diagnostic setting, we processed patient-matched benign and cancer tumor-containing kidney biopsy samples from six nephrectomy patients. Post-extraction biopsy sections were classified by a board-certified pathologist, and representative histology images are shown in Figure 5. We also performed metabolomics analysis on the methanol extracts from these same patient-matched benign and cancer tumor-containing biopsy samples. This represents the first biopsy-derived metabolomic signature of cancer in the human kidney. Sixty-nine metabolites distinguished cancer tumor from benign samples (Additional file 6). These changes are indicative of altered amino acid metabolism, oxidative stress and pyrimidine turnover in the transformed kidney.

**Figure 6:**
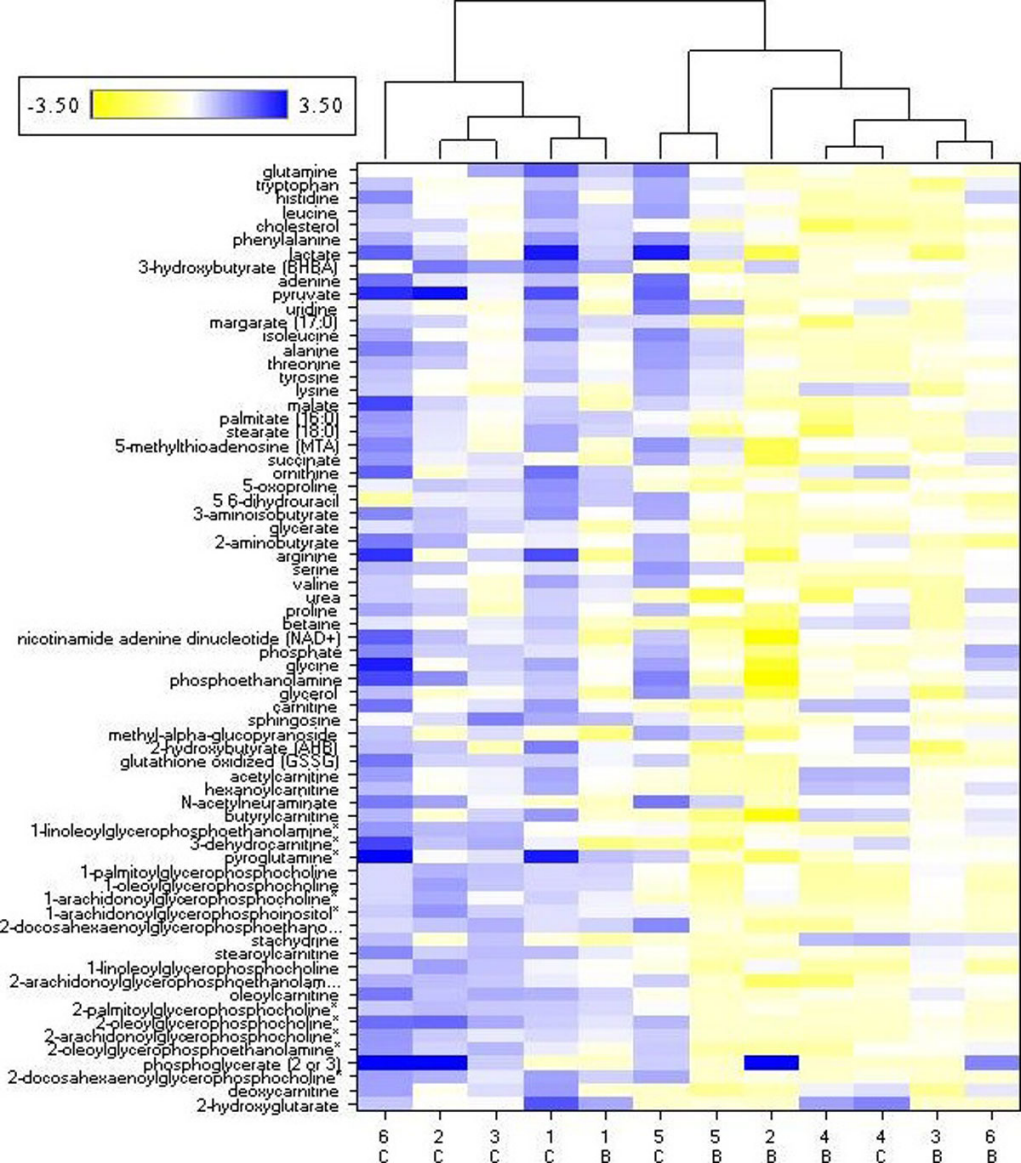
**Cancer tumor and benign kidney samples can be separated using hierarchical cluster analysis**. The 69 metabolites identified as significantly different (*P *≤ 0.05) between cancer tumor and matched benign kidney tissue from six patients were used to generate the cluster based on Euclidean distance. Cancer tumor or benign samples were determined by histopathology evaluation. Metabolites are listed on the y-axis. Each patient is represented by a number (1 to 6) on the x-axis. Cancer tumor (C) and matched benign (B) samples were used for the analysis. Four of six cancer tumor samples were assigned to the same major cluster and five of six benign samples were assigned to the same major cluster.

To determine how the metabolomic profiles of these samples compared with histologic classification, we performed various statistical analyses of the metabolomic data. Hierarchical clustering analysis split the kidney biopsy samples into two major clusters, with one cluster containing four cancer tumor samples and one benign sample, and the other cluster containing five benign and two cancer tumor samples (Figure 6). This analysis suggests that disease-free tissue from the same patient may be an integral component of interpreting metabolomic data from diseased tissue. For example, based upon the metabolomic signature, the histologically benign biopsy from nephrectomy patient 1 was in the same major cluster with cancer tumor biopsies (Figure 6). Similarly, the cancer tumor samples from patients 4 and 5 were in the main cluster with five benign samples. In all three cases, the matched cancer and benign samples from each of those patients group into the same terminal cluster. In contrast, the tumor samples from patients 2, 3, and 6 do not fall into the same major cluster or terminal cluster as the matched benign samples. It is tempting to speculate that these results reflect a difference in the metabolism in the tissue biopsies that may be indicative of the stage or aggressiveness of the cancer tumor. For instance, in patient 1, although the sample appears histologically benign, the metabolomic signature in the benign biopsy may be indicative of a more aggressive cancerous state since it groups with the cancer cluster. In patients 4 and 5, the metabolomic signature for tumor samples groups with the benign cluster, indicating the signature resembles that of benign samples, which could indicate that the cancer was less advanced or less aggressive. Thus, distinct metabolic signature-based groupings of cancer tumor tissue may indicate not only early stage cancer but could distinguish a more aggressive from a less aggressive cancer. More extensive studies with detailed histological assessments would be needed to substantiate these hypotheses.

**Figure 7:**
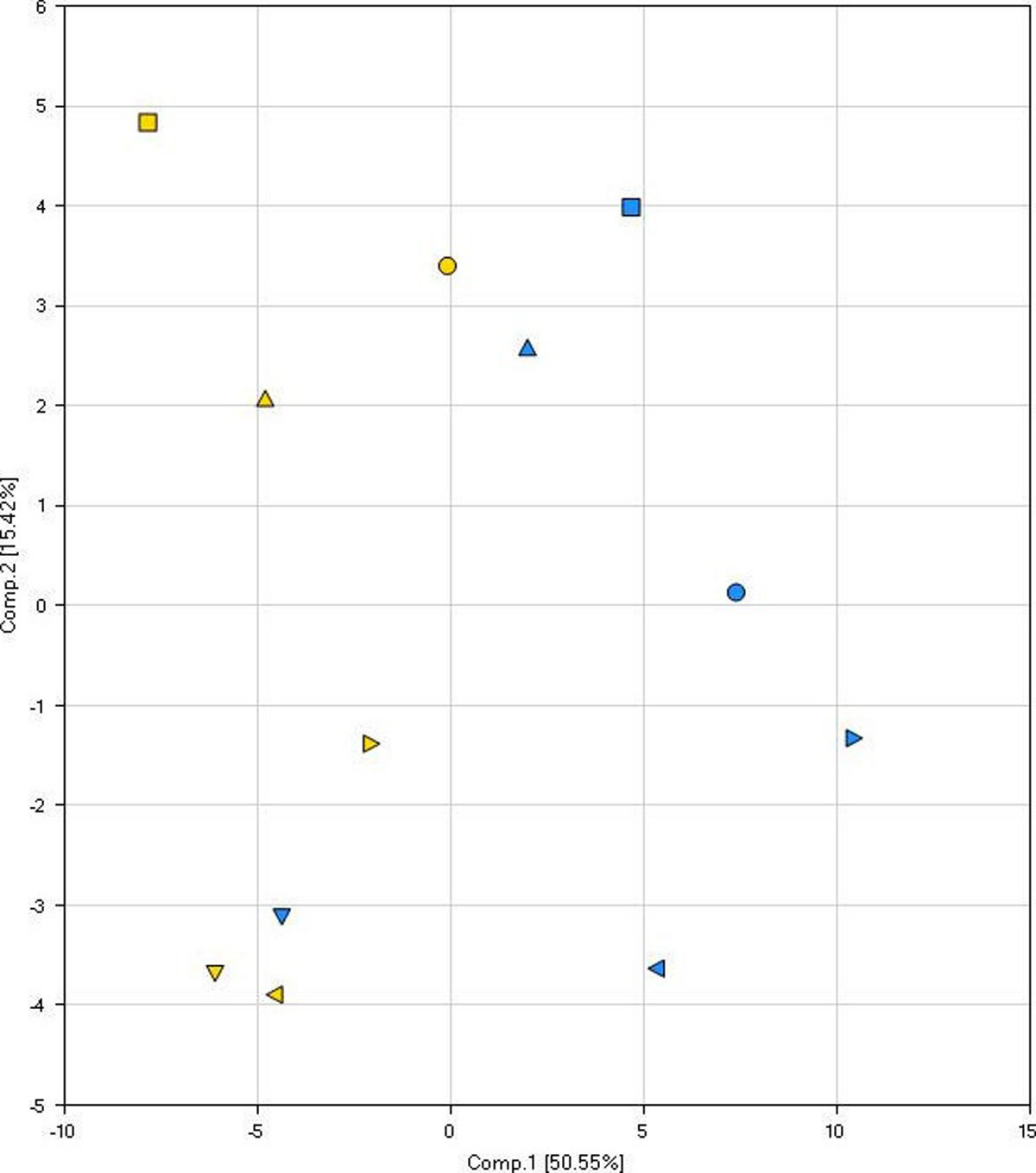
**Principal components analysis of kidney biopsies to distinguish cancer tumor from benign biopsies**. The metabolites identified as significant (*P *≤ 0.05) between cancer tumor-containing and benign kidney biopsies by matched pairs *t*-test were used to construct the principal components analysis. Blue, cancer tumor samples; yellow, benign samples. The six nephrectomy patients are each indicated by a shape: circle, patient 1; square, patient 2; upward triangle, patient 3; downward triangle, patient 4; left pointing triangle, patient 5; right pointing triangle, patient 6.

Random forest analysis classified the kidney biopsy samples based on their metabolomic profiles into cancer tumor or benign groups. All six benign samples were correctly classified and four of the six cancer tumor samples were correct, which gives a predictive accuracy of 83% (Table 4). We also examined these samples using principal components analysis. For five of the six cases examined, there was a significant positive displacement along the first principal component when the cancer tumor biopsy was compared to the patient-matched benign sample (Figure 7). Performing this workflow with a larger cohort is necessary to determine the true clinical effectiveness of the data, but taken together these data suggest that metabolomic profiles obtained using this workflow have the potential to guide and/or augment diagnosis and patient management.

**Table 4:** Classification of kidney biopsy samples based on metabolites extracted from intact biopsies

		Predicted	
		
		Benign	Cancer
Actual	Benign	6	0
	Cancer	2	4

The random forest confusion matrix demonstrates that benign samples can be distinguished from cancer tumor samples by using kidney biopsy samples. The prediction accuracy was 83% and provides an estimate of how accurately new observations can be predicted using the random forest model (for example, whether a sample contains cancer tumor or is benign).

The workflow was also used to assess the metabolomic signature of human prostate cancer and to demonstrate the metabolomic classification of patient-matched benign and cancer tumor-containing prostate biopsy samples from eight prostatectomy patients. Post-extraction biopsy sections were classified by a board-certified pathologist. Amino acid, nucleotide and lipid abundance profiles were markedly altered in the transformed tissues as reported [15]. These results are consistent with previous studies using 100 mg of post-prostatectomy tissue [10], confirming that despite the 20- to 50-fold reduction in material, the metabolic signature is essentially retained.

**Figure 8:**
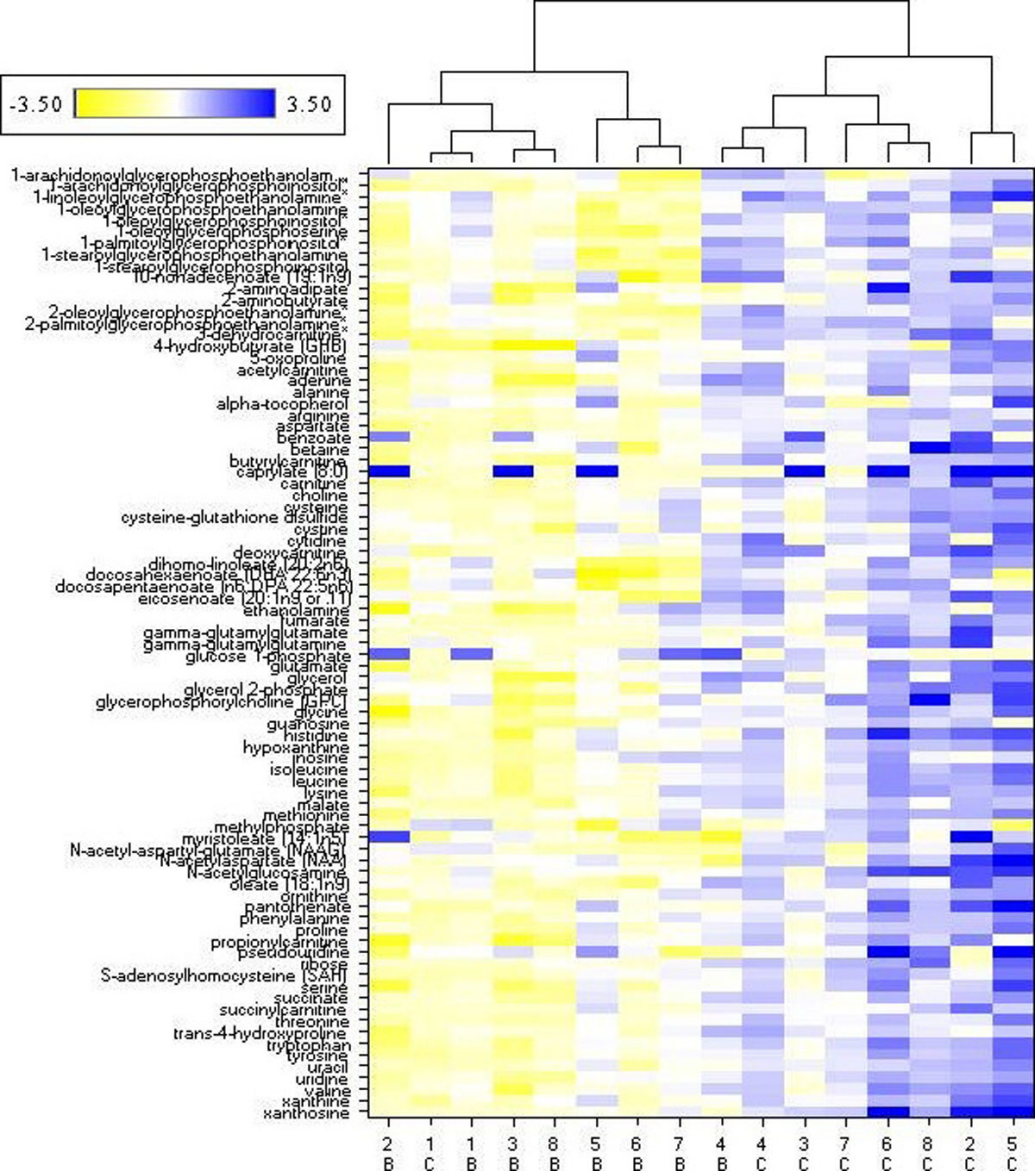
**Hierarchical cluster analysis of cancer tumor and benign prostate samples**. The 83 metabolites determined to be significantly different (*P *< 0.05) between cancer tumor and matched benign tissue from eight patients were used to generate the cluster based on Euclidean distance. Metabolites are listed on the y-axis. Each patient is represented by a number (1 to 8) on the x-axis. Histologically determined cancer tumor (C) and matched benign (B) samples were used for the analysis. Cancer tumor and benign biopsies fall into two major clusters. Seven of eight cancer tumor and seven of eight benign samples clustered as predicted by the histological analysis of the biopsy.

To visualize the relationship between significantly altered metabolites in the prostate biopsy samples, we used hierarchical clustering (Figure 8). The two main clusters separated seven benign and one cancer tumor sample from seven cancer tumor and one benign sample. Matched samples were in the same terminal cluster for patients 1 and 4, with the samples from patient 1 grouped in the benign major cluster and those from patient 4 in the cancer major cluster. As discussed above, these results could indicate differences in cancer stage and aggressiveness for these patients. Random forest analysis classified these samples into cancer tumor or benign groups based on their metabolic profiles with 81% predictive accuracy (Table 5). Analysis of a larger cohort of samples will be necessary to determine whether there are gradations in metabolic profiles across disease severity, but these data demonstrate the sensitivity of metabolomics to further inform histological diagnosis.

**Table 5:** Classification of prostate biopsy samples based on metabolites extracted from intact biopsies

		Predicted	
		
		Benign	Cancer
Actual	Benign	7	1
	Cancer	2	6

The random forest confusion matrix demonstrates that prostate biopsy cancer tumor samples can be distinguished from benign samples. The prediction accuracy was 81% and provides an estimate of how accurately new observations can be predicted using the random forest model (for example, whether a sample contains cancer tumor or is benign).

## Conclusions

We report a workflow that uses histologic and metabolomic analysis on a single core needle biopsy to aid in disease diagnosis in a clinical setting. This method will facilitate the translation of discovery studies into clinically accepted diagnostic tests, and these tests have the potential to bring additional resolving power to current histopathology-based diagnoses. In prostate cancer, for example, a common question is whether a patient with a clinical Gleason Score of 6 or 7 should undergo radical prostatectomy. With 60 to 70% of the approximately 220,000 prostate cancer cases presenting in this Gleason Score range [20], further evidence regarding whether a tumor is aggressive would help inform the physician-patient discussion regarding the choice between expectant management and definitive therapy, including radical radiotherapy or radical surgery. Beyond prostate cancer, there are many other cancers (and potentially other diseases) where biochemical data could supplement histopathology, enhancing diagnostic and prognostic utility. Given the prevalence of cancer in the human population, application of this workflow has the potential to better inform cancer management options for millions of patients worldwide.

## Abbreviations

FISH: fluorescence *in situ *hybridization; GC: gas chromatography; MS: mass spectrometry; MS/MS^2^: tandem mass spectrometry; UHPLC: ultra high-performance liquid chromatography.

## Competing interests

MVB, JEM, PRG, MVM, and KAL are employees of Metabolon, Inc. DAT is a stockholder in Provia Biologics, a licensee of intellectual property from Eastern Virginia Medical School for the method. EMS has no disclosures. The authors have no other relevant affiliations or financial involvement with any organization or entity with a financial interest in or financial conflict with the subject matter or materials discussed in the manuscript apart from those disclosed. No writing assistance was utilized in the production of this manuscript.

## Authors' contributions

MVB designed and carried out the experiments, analyzed the data, and drafted the manuscript. JEM analyzed the data and helped to draft the manuscript. PRG performed the statistical analysis. EMS assisted with sample procurement and refined the method for histologic processing of biopsy samples. MVM participated in the design of the study. DAT conceived of the study, performed histological analysis, and helped to draft the manuscript. KAL conceived of the study, participated in its design and coordination and helped to draft the manuscript. All authors read and approved the final manuscript for publication.

## Supplementary Material

Additional file 1 **Number and identity of metabolites obtained with the intact biopsy extraction method and the standard ground tissue extraction method**. We used 70% methanol for intact biopsy extraction. The total number of metabolites detected with each method is shown in the rectangles at the bottom. The Venn diagram illustrates the overlap in the identity of metabolites detected using each sampling strategy (30 mg tissue, intact biopsy, and ground biopsy). The vast majority of metabolites (273) can be detected using any of the methods. Click here for file

Additional file 2 **Efficiency of intact biopsy extraction**. Heatmap view of metabolites remaining in the biopsy following intact biopsy extraction. Biopsy samples were methanol extracted, then the intact biopsy tissue was removed, ground and re-extracted to determine the efficiency of the metabolite extraction using this method. Most metabolites (156 of 299) were fully extracted from the intact biopsy and the vast majority of metabolites detectable in the secondary extraction (132 of the 143) were significantly reduced, typically by more than 80%. Colored cells indicate statistically significant changes, blue indicates metabolites that were significantly higher (N = 1), yellow indicates metabolites that were significantly lower (N = 131) in the secondary extraction of the ground tissue relative to the intact biopsy extract. Click here for file

Additional file 3 **Incubation time course of metabolites detected in kidney tissues using intact biopsy extraction method**. Biopsy samples from normal human kidney tissue were incubated in 80% methanol for 0.5 h, 1 h, 4 h, 8 h, 16 h, 24 h, and 48 h. The extracts were processed for metabolomics analysis. The identity of metabolites detected following each incubation time are listed and grouped by biochemical pathway. An 'X' indicates that the biochemical was detected. Click here for file

Additional file 4 **Metabolites detected in various tissues**. Metabolites detected using the intact biopsy method are listed and grouped by biochemical pathway. Adrenal gland, colon, lung, muscle, pancreas, small intestine, spleen, and stomach tissue were obtained with consent from beating heart donors according to guidelines. Prostate metabolome is reported for benign prostate tissue from prostatectomy patients obtained with consent according to guidelines. An 'X' indicates that the biochemical was detected. Click here for file

Additional file 5 **Representative histology images of tissue biopsies show tissue architecture is retained**. Adrenal gland, colon, lung, muscle, small intestine, spleen, and stomach tissue biopsies were processed using the intact biopsy workflow and stained with hematoxylin and eosin. The tissues were obtained with consent from beating heart donors according to guidelines. Click here for file

Additional file 6 **Metabolites significantly changed in cancer tumor compared to benign kidney biopsies**. Heatmap view of metabolites significantly altered when comparing kidney cancer tumor-containing biopsies to matched benign biopsies. All samples were processed with the intact biopsy workflow. Matched pairs *t*-test was used to identify 69 biochemicals meeting the significance criteria (*P *≤ 0.05, q ≤ 0.1). Click here for file
