# Supplementary material for: Cancer detection and biopsy classification using concurrent histopathological and metabolomic analysis of core biopsies
Source: Genome Med. 2012 Apr 30;4(4):33. doi: 10.1186/gm332 (PMC3446261; doi:10.1186/gm332)
Supplement: Additional file 1 — Number and identity of metabolites obtained with the intact biopsy extraction method and the standard ground tissue extraction method. We used 70% methanol for intact biopsy extraction. The total number of metabolites detected with each method is shown in the rectangles at the bottom. The Venn diagram illustrates the overlap in the identity of metabolites detected using each sampling strategy (30 mg tissue, intact biopsy, and ground biopsy). The vast majority of metabolites (273) can be detected using any of the methods. [file gm332-S1.ppt]

## Slide 1
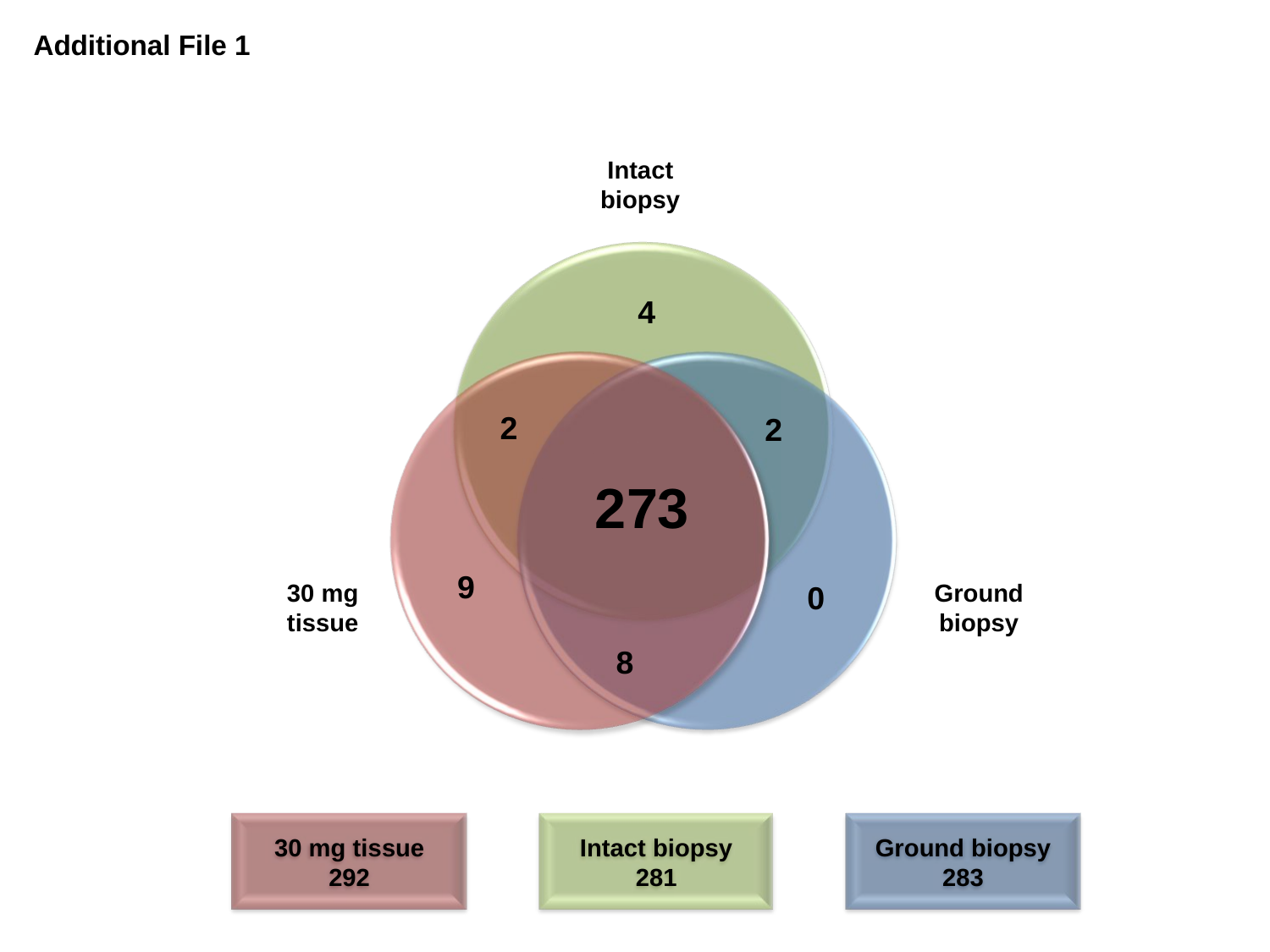

Additional File 1
Intact biopsy
4
2
2
273
9
30 mg tissue
0
Ground biopsy
8
30 mg tissue
292
Intact biopsy
281
Ground biopsy
283
