# Supplementary figures and images for: Cancer detection and biopsy classification using concurrent histopathological and metabolomic analysis of core biopsies
Source: Genome Med. 2012 Apr 30;4(4):33. doi: 10.1186/gm332 (PMC3446261; doi:10.1186/gm332)

## Slide 1
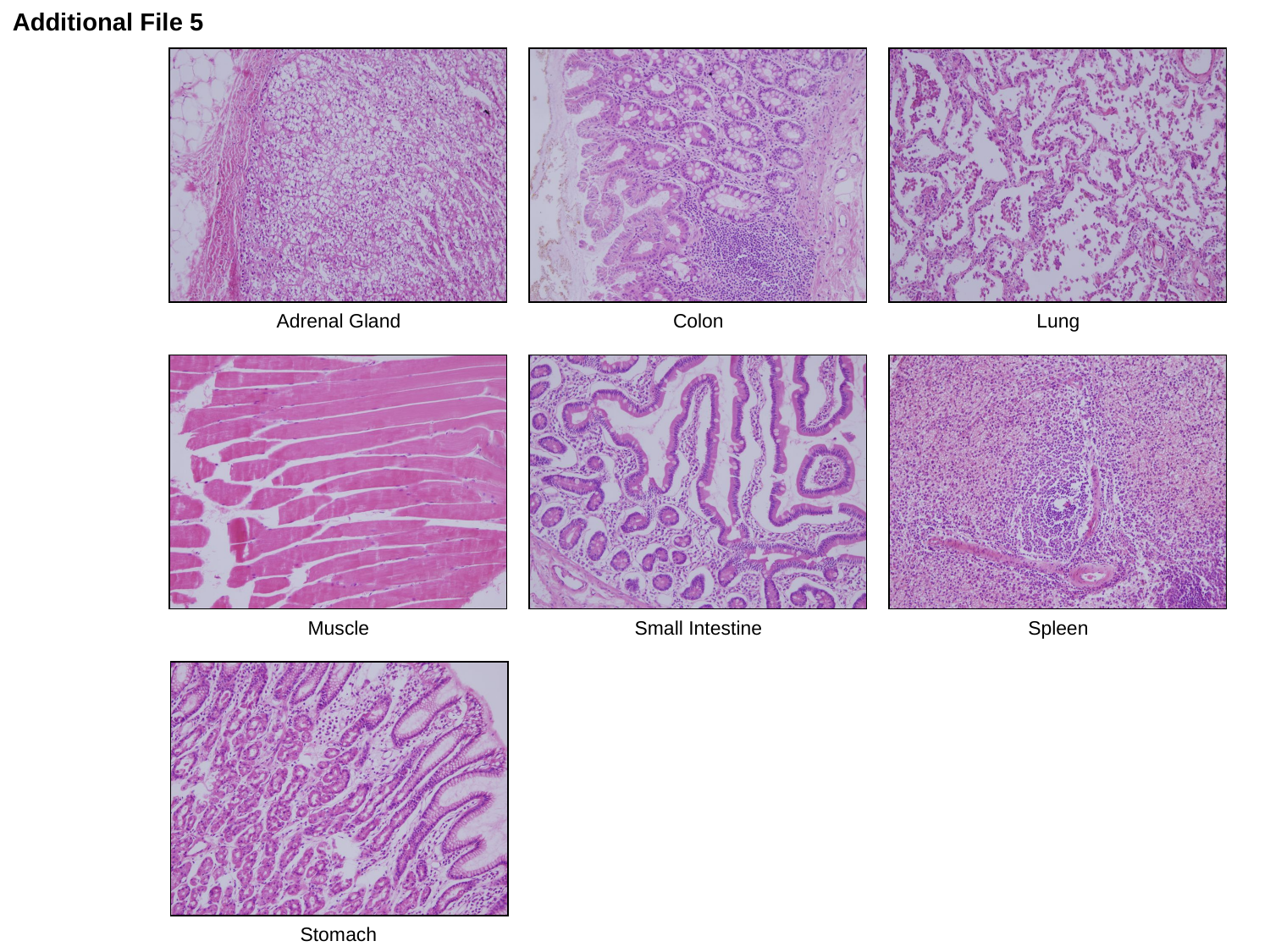

Additional File 5
Adrenal Gland
Colon
Lung
Muscle
Small Intestine
Spleen
Stomach

Supplement: Additional file 5 — Representative histology images of tissue biopsies show tissue architecture is retained. Adrenal gland, colon, lung, muscle, small intestine, spleen, and stomach tissue biopsies were processed using the intact biopsy workflow and stained with hematoxylin and eosin. The tissues were obtained with consent from beating heart donors according to guidelines. [file gm332-S5.ppt]
